# Apoptosis-induced activation of HIV-1 in latently infected cell lines

**DOI:** 10.1186/s12977-015-0169-1

**Published:** 2015-05-16

**Authors:** Sohrab Z. Khan, Nicholas Hand, Steven L. Zeichner

**Affiliations:** Center for Cancer and Immunology Research, Children’s Research Institute, Children’s National Medical Center, Washington, DC USA; Department of Microbiology, Immunology, and Tropical Medicine, The George Washington University School of Medicine, Washington, DC USA; Department of Pediatrics, The George Washington University, School of Medicine, Washington, DC USA

**Keywords:** Latency, Apoptosis, Caspase, Activation, HIV

## Abstract

**Background:**

Despite much work, safe and effective approaches to attack and deplete the long-lived reservoir of cells latently infected with HIV-1 remain an elusive goal. Patients infected with HIV-1 treated with cytotoxic agents or bone marrow transplantation can experience decreases in the reservoir of HIV-1 latently infected cells. Other viruses capable of long-term latency, such as herpesviruses, can sense host cell apoptosis and respond by initiating replication. These observations suggest that other viruses capable of long-term latency, like HIV-1, might also sense when its host cell is about to undergo apoptosis and respond by initiating replication.

**Results:**

Pro-monocytic (U1) and lymphoid (ACH-2) HIV-1 persistently infected cell lines were treated with cytotoxic drugs – doxorubicin, etoposide, fludarabine phosphate, or vincristine – and activation of latent HIV-1 was evaluated using assays for HIV-1 RNA and p24 production. Both cell lines showed dose-dependent increases in apoptosis and associated HIV-1 activation following exposure to the cytotoxic agents. Pretreatment of the cells with the pan-caspase inhibitor Z-VAD-FMK prior to exposure to the cytotoxic agents inhibited apoptosis and viral activation. Direct exposure of the latently infected cell lines to activated caspases also induced viral replication. HIV-1 virions produced in association with host cell apoptosis were infectious.

**Conclusions:**

The results indicate that latent HIV-1 can sense when its host cell is undergoing apoptosis and responds by completing its replication cycle. The results may help explain why patients treated with cytotoxic regimens for bone marrow transplantation showed reductions in the reservoir of latently infected cells. The results also suggest that the mechanisms that HIV-1 uses to sense and respond to host cell apoptosis signals may represent helpful new targets for approaches to attack and deplete the long-lived reservoir of cells latently infected with HIV-1.

## Background

Effective antiretroviral therapy (ART) can reduce HIV-1 circulating in peripheral blood to below the limits of detection. However, ART cannot completely eradicate HIV-1 because of the persistent reservoir of latently infected cells [1–4], and reviewed in [5–7]. Much work has recently focused on finding ways to deplete or eliminate the long-lived reservoir of latently infected cells. One approach that has gained attention is termed “shock (or kick) and kill,” which aims to deplete the reservoir by activating latent HIV while using ART to prevent infection of new cells (reviewed in [8–11]). However, it has been challenging to develop safe and effective agents that can activate HIV-1 in all latently infected cells. Agents that have been studied include those acting through the NF-κB pathway [12–16] and agents that activate HIV-1 by altering the epigenetic environment of the integrated provirus. Epigenetic agents that have been studied as HIV activators include DNA methylation inhibitors [17–19], histone deacetylase inhibitors (HDACis) [20–24], disulfiram [25] and vorinostat [26–28]. Although such agents can activate HIV-1 *in vivo*, they fail to completely purge latent reservoirs from the infected individuals [29]. Some reports suggest that HDACis are less able to activate HIV in a primary cell latency model [30] or in resting CD4+ T cells from ART-treated HIV-1-infected patients [31] compared to infected transformed cell lines. Other efforts have gone into developing less toxic latency reversing agents that act as inducers for the protein kinase C (PKC) signaling and NF-κB pathways, such as prostratin [15, 32, 33] and bryostatin-1 [29, 34]. However, there are still important concerns with these compounds because PKC signaling has widespread effects on host cell metabolism, so agents that target PKC signaling may raise regulatory concerns (reviewed in [35, 36]).

While several approaches aimed at activating latent HIV-1 have been developed, none of them have proven effective at activating all latent viruses. We previously studied the ability of different activating agents to induce HIV-1 replication in several distinct cell line models of latent infection, which may reflect some of the diversity that exists among latently infected cells *in vivo*, and found that agents that activate HIV-1 in some of the cell lines could not activate the virus in other cells lines and that some agents showed antagonistic effects in some model cell lines [37].

Recent work showed that the reservoir of cells latently infected with HIV-1 may be even more difficult to attack than was previously appreciated. For example, a study showed that T-cell activation does not induce all of the functional latent provirus present, and a significant proportion of these non-induced proviruses are replication-competent [38]. If agents are unable to activate all latent HIV-1 in the reservoir, much of the provirus that remains may be capable of reinitiating and sustaining infection. In the “shock and kill” approach essentially all HIV-1 in the latent reservoir must be eradicated to effect a cure.

While agents specifically designed to activate HIV-1 have proven to be incompletely effective, other therapeutic interventions, involving cytotoxic chemotherapy and bone marrow transplantation (BMT), appear to have been relatively more effective at attacking and depleting the HIV-1 latent reservoir. These examples include the only patient known to have been cured of HIV-1 infection, and other patients that while not cured nevertheless experienced substantial reductions in the reservoir [39–42], although for these patients rebound viremia was observed 15 weeks after treatment interruption [43].

In these studies, in which patients were treated with bone marrow transplantation with continued antiretroviral therapy or using a donor who had the Δ32CCR5 mutation, it is understandable why no new cells were infected, but it is not clear how and why BMT or associated cytotoxic conditioning regimen eliminated or significantly reduced HIV-1 latent reservoirs in these patients. One possible, but unlikely explanation is that the cytotoxic agents simply killed all the latently infected cells. Another possible explanation for the reservoir reductions seen in the bone marrow transplantation patients is that the latently infected cells were eliminated by a phenomenon analogous to the well-known graft *vs.* tumor effect that significantly contributes to the cancer cures observed after bone marrow transplantation [44, 45]. However, HIV-1 patients treated with bone marrow transplantation for lymphoma showed only a weak anti-HIV-1 cellular immune response [43]. The precise mechanisms responsible for the HIV reservoir reductions seen in association with bone marrow transplantation remain unclear.

HIV-1, like many other viruses, has evolved ways to inhibit host cell apoptosis [46–51], an important way for the virus to enhance its replication when host cells initiate the apoptotic program as a way of limiting replication within the host. When herpesviruses fail to prevent the host cell from undergoing apoptosis, they apparently have another strategy to try to ensure production of some progeny virions. We recently found that when KSHV [52], HHV6A, HHV6B, HHV7 and EBV [53] detect that the host cell is undergoing apoptosis, they adopt an emergency escape mechanism, an Alternative Replication Program (ARP), a process that leads to the rapid production of large amounts of virus with decreased infectivity. Caspase-3 is necessary and sufficient to initiate the ARP. The Roizman lab showed that herpes simplex virus type 1 (HSV-1) has a similar alternative replication program when it senses that its host cell is about to undergo apoptosis [54, 55]. The existence of an apoptosis-triggered ARP makes evolutionary sense. Without an apoptosis-triggered ARP, once the apoptotic program begins, the host cell would die before any progeny virus was produced. An apoptosis-triggered ARP would therefore appear to be a helpful survival strategy for any virus capable of long-term latency. Although herpesviruses and retroviruses are members of completely different Families, any virus capable of long term latent infection should still be subject to the same evolutionary pressures. An analogous apoptosis-triggered replication program could help provide an explanation for the reductions in latent HIV-1 reservoirs observed in patients treated with cytotoxic agents during bone marrow transplantation. Apoptotic signals sensed by the virus would then trigger viral replication, leading to a reduction in the viral latent reservoir, when the patients are also treated with antiviral agents or transplanted with cells incapable of being infected with HIV-1, in a process beyond those attributed to other mechanisms.

To explore the hypothesis that HIV-1 can sense and respond to host cell apoptosis, we tested the ability of HIV-1 latently infected cell lines to initiate viral replication in response to cytotoxic agents, and directly to activated-caspases. We found that apoptosis triggered by cytotoxic drugs triggered HIV-1 replication, and that inhibiting apoptosis with caspase inhibitors led to a reduction in viral replication. The process produced infectious virions, had kinetics that differed from the kinetics observed following activation with conventional agents, and occurred in latently infected cells arrested in G_1_, in addition to actively replicating cells. The presence of activated caspases was directly associated with the initiation of viral replication, suggesting that HIV-1 can sense host cell apoptosis and respond by initiating replication.

## Results

### Apoptosis triggers HIV-1 activation in latently-infected cells

**Fig. 1:**
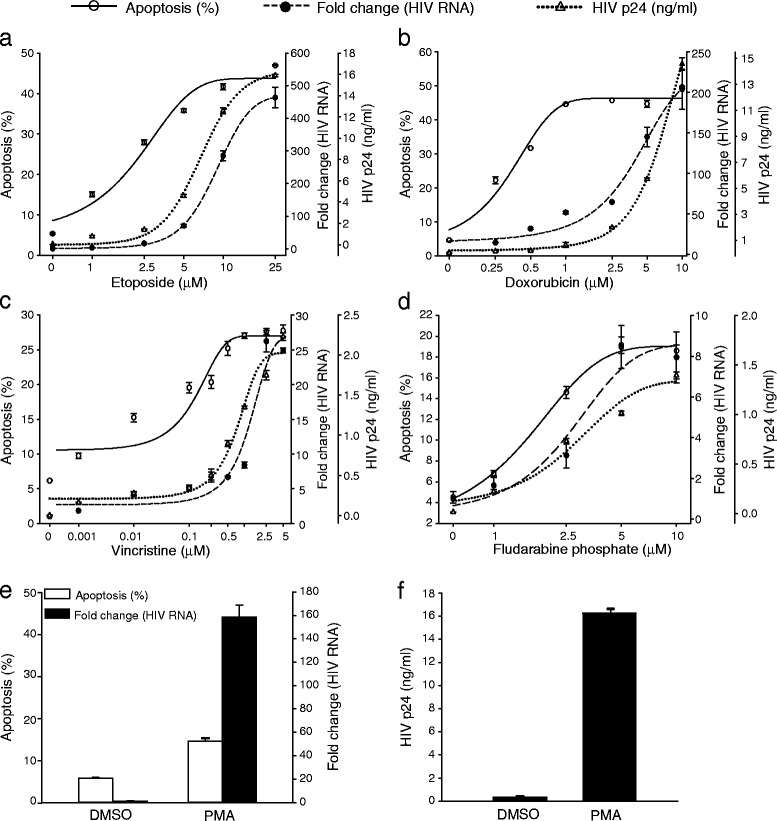
Cytotoxic drugs induce apoptosis and HIV-1 replication in U1 cells. The cells were treated with (**a**) etoposide (0–25 μM), (**b**) doxorubicin (0–10 μM), (**c**) vincristine (0–5 μM) and (**d**) fludarabine phosphate (0–10 μM). At 36 h post treatment, apoptosis was assessed by Annexin V staining (solid line) and HIV-1 activation was determined by qRT-PCR for unspliced viral RNA (dashed line) and ELISA for HIV-1 p24 viral antigen (dotted line) in culture supernatant. (**e**) PMA (25 ng/ml) was used as a positive control, bar graph shows apoptosis (%, white bars) and fold increase in HIV-1 unspliced RNA production (black bars). (**f**) HIV-1 production after PMA treatment was determined by ELISA for p24 antigen in culture supernatant from PMA treated U1 cells. The data represented are the mean ± standard deviation (n = 3)

To examine the effects of cytotoxic drug-induced apoptosis on latent HIV-1, we studied pro-monocytic U1 cells latently infected with HIV-1. U1 cells contain two integrated copies of the viral genome and, under unstimulated conditions, express low levels of viral transcripts encoding Tat, Rev, and Nef, but little or no full-length viral RNA [56, 57]. Cells were treated with four pro-apoptotic cytotoxic agents frequently used for cancer chemotherapy and in bone marrow transplant conditioning regimens: etoposide (Fig. 1a), doxorubicin (Fig. 1b), vincristine (Fig. 1c), and fludarabine phosphate (Fig. 1d). In addition to the cytotoxic agents, aliquots of cells were also incubated with DMSO (at a concentration equal to the one the cells were exposed to after addition of the cytotoxic agents) as negative control and treated with PMA (25 ng/ml) as a positive control for viral induction through a PKC-related pathway (Fig. 1e and f). Cells were collected 36 h post treatment and apoptosis was evaluated by flow cytometry for Annexin-V^+^/7-AAD staining. HIV-1 activation was monitored by p24-capture ELISA and qRT-PCR for unspliced HIV-1 RNA. Exposure to each of the four cytotoxic agents caused dose-dependent increases in apoptosis, with concurrent production of viral RNA within the cells and p24 in culture supernatant (Fig. 1 a–d). Fig. 1 show that with increasing concentration of each drug, the number of Annexin-V^+^ cells significantly increased with simultaneous production of viral RNA and p24 production. Fig. 1 e and f show that treatment of the cells with PMA, which activates HIV-1 through the classical NF-κB pathway, activates HIV-1 in these cells, and that DMSO, the solvent in which we dissolved the cytotoxic agents did not activate HIV-1. Some of the cytotoxic agents, etoposide and doxorubicin, produced larger fold-activation levels than the PMA positive control inducer, while others were less effective (vincristine and fludarabine phosphate), but all were highly activating, with the least potent activator, fludarabine phosphate, producing a ~10-fold increase in HIV RNA.

**Fig. 2:**
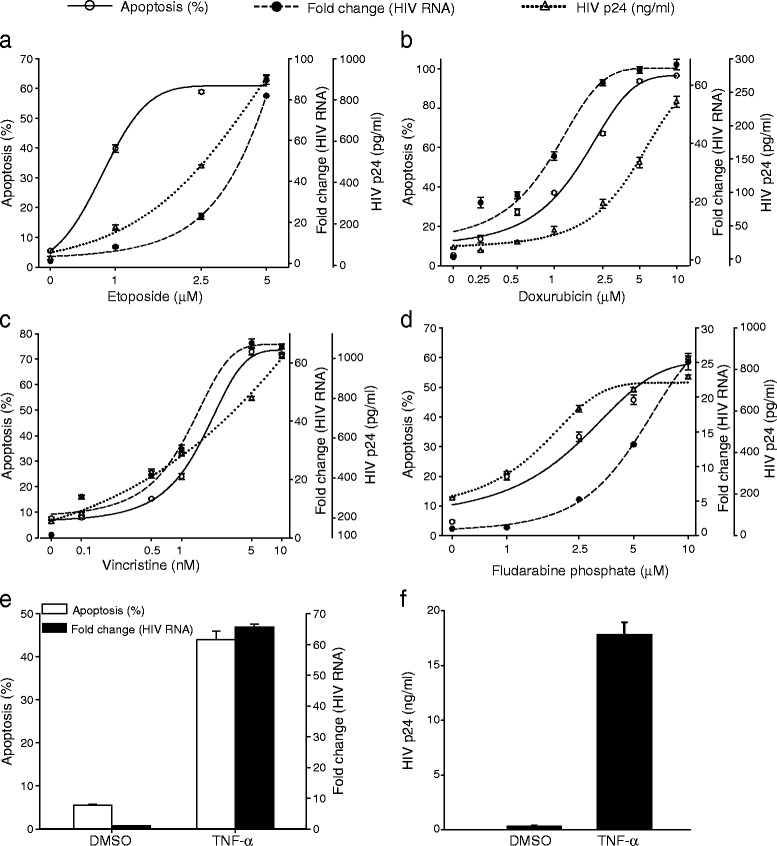
Induction of HIV-1 replication by apoptosis in ACH-2 cells. 36 h post treatment with (**a**) etoposide (0–5 μM), (**b**) doxorubicin (0–10 μM), (**c**) vincristine (0–10 nM) and (**d**) fludarabine phosphate (0–10 μM), apoptosis was assessed by Annexin-V staining (solid line). HIV-1 activation was measured as fold change (compared to control DMSO treated cells) in accumulation of unspliced HIV-1 RNA by qRT-PCR (dashed line). HIV-1 production after drug exposure was determined using ELISA for HIV-1 p24 (dotted line). (**e**) For a positive control, cells were treated with TNF-α (20 ng/ml) and HIV-1 activation was measured by qRT-PCR as mentioned above. (**f**) Virus production from ACH-2 treated with TNF-α was determined by ELISA for HIV-1 p24 antigen. The data represented are the mean ± standard deviation (n = 3)

**Fig. 3:**
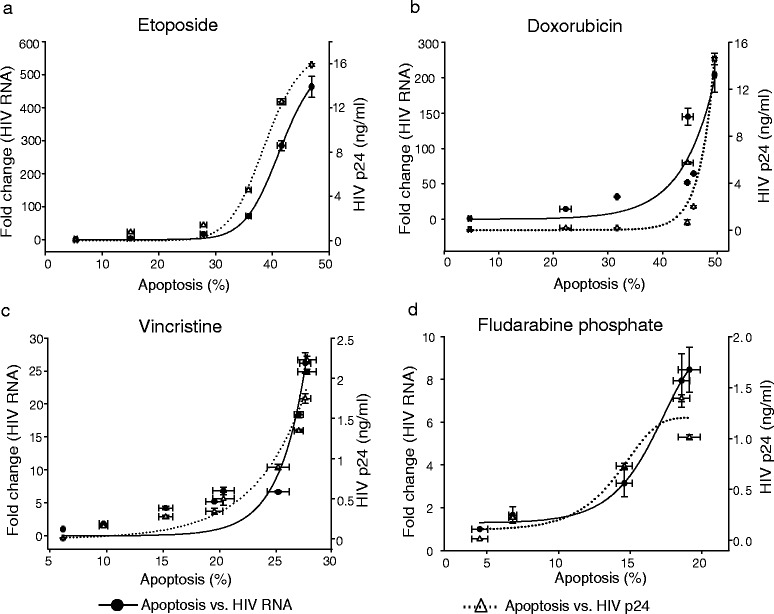
HIV-1 activation in response to apoptosis induction in U1 cells. The figure shows the relationship between HIV-1 replication and apoptosis triggered by cytotoxic drugs: (**a**) etoposide, (**b**) doxorubicin, (**c**) vincristine and (**d**) fludarabine phosphate. Apoptosis and HIV-1 activation was assessed as mentioned in Fig. 1 and Fig. 2. Spearman correlation coefficient (ρ) for apoptosis *vs.* HIV-1 activation for all the drugs was ≥ 0.9. Values represented are the mean ± SD (n = 3)

**Fig. 4:**
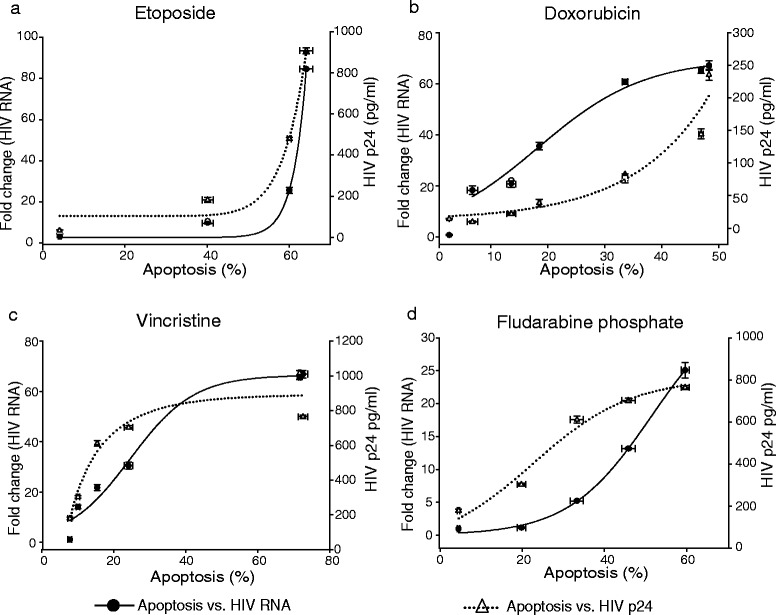
Relationship between apoptosis and HIV-1 activation in ACH-2 cells. The figure shows the relationship between HIV-1 replication and apoptosis triggered by cytotoxic drugs: etoposide (**a**) etoposide, (**b**) doxorubicin, (**c**) vincristine and (**d**) fludarabine phosphate. Results are pooled from three independent experiments and are shown as the mean ± SD of three independent experiments. The Spearman correlation coefficient (ρ) for apoptosis *vs.* HIV-1 activation for all the drugs was ≥ 0.86

To confirm that apoptosis activates latent HIV-1 in other cell types, we studied a lymphocytoid HIV-1 latently infected cell line, ACH-2, [58]. HIV-1 in ACH-2 cells has a defect in the Tat responsive element (TAR) due to a single point mutation in the loop of the TAR hairpin [59]. ACH-2 cells were treated with the cytotoxic drugs and harvested 36 h post treatment. The cells were also incubated with TNF-α (20 ng/ml) and DMSO to serve as positive and negative controls (Fig. 2e and f). We found a similar dose-dependent pattern of apoptosis-mediated HIV-1 activation as observed in U1 cells (Fig. 1), when ACH-2 cells were treated with cytotoxic agents; etoposide (Fig. 2a), doxorubicin (Fig. 2b), vincristine (Fig. 2c), and fludarabine phosphate (Fig. 2d). The increase in apoptosis was associated with HIV-1 activation at the RNA and protein levels and was observed for all cytotoxic drugs. ACH-2 cells were more sensitive to etoposide and vincristine-mediated cell death (Fig. 2a and c) compared to U1 cells (Fig. 1a and c). It is interesting to note that apoptosis induced by different drugs produced different levels of HIV-1 activation. While a detailed explanation of the mechanisms underlying these differences must await future work, the different cytotoxic agents induce apoptosis through different mechanisms, induce apoptosis at different concentrations, and the latently infected cells have different sensitivity to apoptotic agents [60, 61]. In spite of observed difference in the apoptosis dose-response in the different cells, apoptosis was consistently associated with HIV-1 activation. Taken together, these results suggest that apoptosis broadly triggers HIV-1 replication in both HIV-1 latently infected cell line model systems. When we reanalyzed the data, examining HIV activation as a function of apoptosis, we found a clear correlation, with large increases in HIV-1 activation observed when between 30 and 50 % of cells were apoptotic, depending on the cytotoxic agent and cell line. We found that there was a significant association (Spearman’s coefficient (ρ) ranged from 0.88 to 0.99) for all pro-apoptotic chemotherapy agents used to treat U1 (Fig. 3) and ACH-2 cells (Fig. 4).

**Fig. 5:**
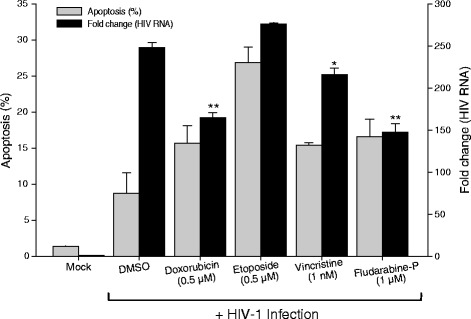
Effect of apoptosis induction on active HIV-1 replication. Jurkat cells were infected with HIV-1. The infected cells were treated with apoptosis inducers: doxorubicin (0.5 μM), etoposide (0.5 μM), vinicristine (1 nM) and fludarabine phosphate (1 μM). Apoptosis (grey bars) was assessed by Annexin-V staining and HIV-1 unspliced RNA was assessed by qRT-PCR (black bars) were assessed 48 h post infection. Values presented as mean ± SD, with statistically significant differences indicated by asterisks (**p ≤ 0.001 and *p ≤ 0.05 vs DMSO-treated cells)

These data indicate that cytotoxic drug induced-apoptosis can reactivate latent HIV-1 in ACH-2 and U1 cells. It is possible that apoptosis may also affect active HIV-1 replication. To examine this, we infected Jurkat T-cells with HIV-1, and then treated the cells with cytotoxic drugs. We observed no significant increase in HIV-1 RNA in the cells treated with apoptosis inducers compared with infected cells that were not treated with apoptotic agents (Fig. 5). Rather, we observed significant reductions in the HIV RNA produced by the cells treated with the cytotoxic agents. The data suggest that while treatment with agents that induce apoptosis can trigger activation of latent HIV, treatment with agents that induce apoptosis during active HIV replication does not enhance viral replication, and if anything works against effective viral replication, an observation consistent with the observations that HIV-1 has several mechanisms aimed at preventing host cell apoptosis during active viral replication.

### HIV-1 reactivation in G_1_-phase resting cells

**Fig. 6:**
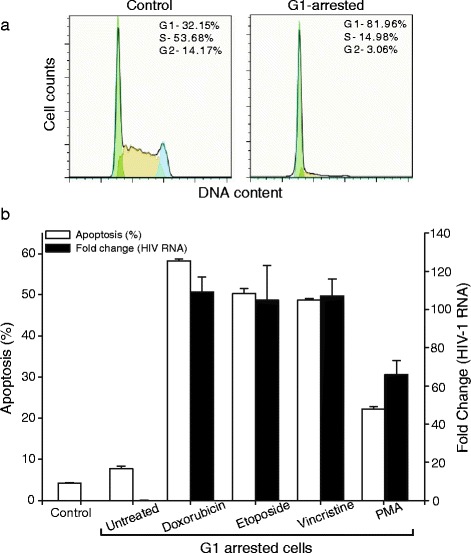
Apoptosis-mediated HIV activation in G_1_-arrested cells. (**a**) Synchronization of U1 culture using simvastatin to enrich the cells in G_1_-phase. (**b**) Apoptosis in synchronized cells was induced by doxorubicin (2.5 μM), etoposide (5 μM) or vincristine (5 μM). Cells induced with PMA were used as positive control for HIV-1 activation. Apoptosis (white bars) was assessed by Annexin-V staining and HIV activation was measured by qPCR for unspliced HIV-1 RNA

The cells from the monocyte-macrophage lineage and resting CD4+ T cells constitute the bulk of the HIV-1 latent reservoir, with resting CD4+ T cells constituting a major part. To assess the ability of apoptosis to induce HIV activation in quiescent or G_1_-arrested cells we studied activation in synchronized U1 cells. The percentage of cells in G_1_-phase was substantially increased (to ~81 % from ~32 %) by simvastatin treatment prior to induction of apoptosis by cytotoxic drugs (Fig. 6a). Drug-induced apoptosis strongly activated HIV expression in the G_1_-arrested cells, and showed a more pronounced increase in HIV-1 activation compared to conventional activation by PMA (Fig. 6b). Apoptosis can apparently strongly activate HIV-1 in both actively dividing cells and in G_1_-arrested cells.

### Kinetics of HIV-1 activation in response to apoptosis induction

**Fig. 7:**
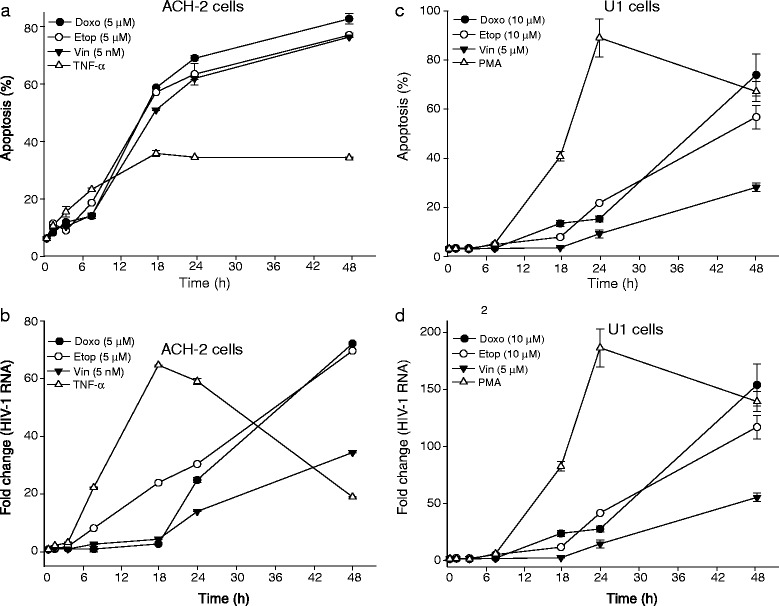
Apoptosis induced HIV-1 reactivation kinetics. The time course of HIV-1 activation study was determined in ACH-2 cells (**a** and **b**) and U1 cells (**c** and **d**) after treatment with apoptosis inducing agent. The cells were harvested at the indicated time-points (0, 2, 4, 8, 18, 24, and 48 h) and assayed for apoptosis (**a** and **c**) by Annexin-V staining and HIV-1 activation was quantitated by RT-qPCR for HIV-1 unspliced RNA (**b** and **d**). Cells treated with PMA (for U1 cells) and TNF-α (for ACH-2) were used as positive controls

HIV-1 replication triggered by cytotoxic agents is tightly linked to apoptosis (Figs. 3 and 4). To study the kinetics of HIV activation following induction of apoptosis, we performed a time course experiment in which we determined HIV-1 expression at serial times after treatment with cytotoxic drugs (Fig. 7). We observed that apoptosis (Fig. 7a and c) was significantly increased 8 h after treatment in both the ACH-2 and U1 cells. However, the induction of HIV-1 expression (Fig. 7b and d) was evident only after 18 h. The conventional NF-κB pathway activated by PMA or TNF-α showed significant induction of HIV-1 expression at 8 h. This data suggests that HIV-1 activation induced by apoptosis has slower kinetics than that elicited by TNF-α or PMA, which may have implications for the mechanisms involved in mediating apoptosis-triggered HIV replication.

### Apoptosis-mediated HIV-1 activation produces infectious HIV-1 virions

**Fig. 8:**
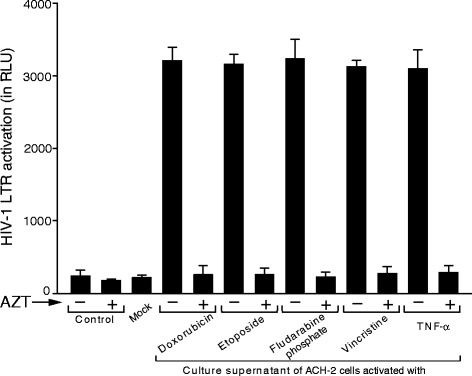
Infectious virion production after induction of replication by apoptosis. The infectivity of virions produced *via* apoptosis-mediated HIV-1 activation was determined using luciferase assay by exposing 1G5 cells to supernatants from cells treated with cytotoxic agents. Equal amounts (200 ng) of virus-containing supernatants, as determined by p24 assays were added to the 1G5 cells (with or without AZT, 5 μM) and luciferase activity determined. Infectious virus was produced following induction of replication with both conventional induction and apoptosis induction. For mock infections, 1G5 cells were incubated with culture supernatant of Jurkat cells treated with TNF-α. Values represented as mean ± SD (n = 3)

It is possible that the pro-apoptotic treatments merely led to the non-productive synthesis of HIV-1 RNA and protein, without the production of infectious virions. To determine whether HIV-1 activation by the cytotoxic treatments actually produced infectious virions, we used the reporter cell line 1G5, a Jurkat derivative containing a stably integrated HIV-LTR-luciferase construct [62]. The IG5 cell line enables evaluation of infectious virus because it has an HIV LTR that controls expression of a luciferase reporter gene. When HIV infects the 1G5 cells, after reverse transcription and integration, production of Tat by the infecting virus transactivates the luciferase reporter. We tested the infectivity of virions released from ACH-2 cells treated with cytotoxic drugs or TNF-α as a positive control. (After activation, U1 cells do not produce virus capable of infecting other cells [63, 64].) We also treated the 1G5 cells infected with virus produced by the ACH-2 cells exposed to the cytotoxic agents with zidovudine (AZT) to confirm that the activation of the luciferase reporter was due to HIV replication. We added aliquots of the supernatants from the cells exposed to the cytotoxic agents or a positive control inducer, TNF-α, normalized for p24 content, and assayed for luciferase activity after 48 h. We found that the 1G5 cells showed large increases in luciferase activity when exposed to supernatants from cells treated with the cytotoxic agents or the positive control activator, TNF-α (Fig. 8). The amounts of luciferase produced by the cells were approximately equal, suggesting that the specific infectivity of the virions produced after activation by the different agents was approximately equal and approximately equal to the infectivity of virus produced through the conventional pathway. AZT treatment of the 1G5 cells completely inhibited luciferase expression, suggesting that the observed increase in luciferase activity is due infectious virions. A minimal signal was obtained in mock treated cells that were exposed to culture supernatant from Jurkat cells treated with TNF-α (Fig. 8). This observation suggests that infectious virions produced the large increases in luciferase activity, and that the increases in luciferase activity were not due to residual cytotoxic drugs or TNF-α or factors released from activated cells. Overall, the data suggests that apoptosis activates latent HIV-1, which results in the production of infectious virions with specific infectivities that similar to virions produced *via* a conventional pathway.

### Activation of HIV-1 replication by cytotoxic drugs depends on caspase activity

**Fig. 9:**
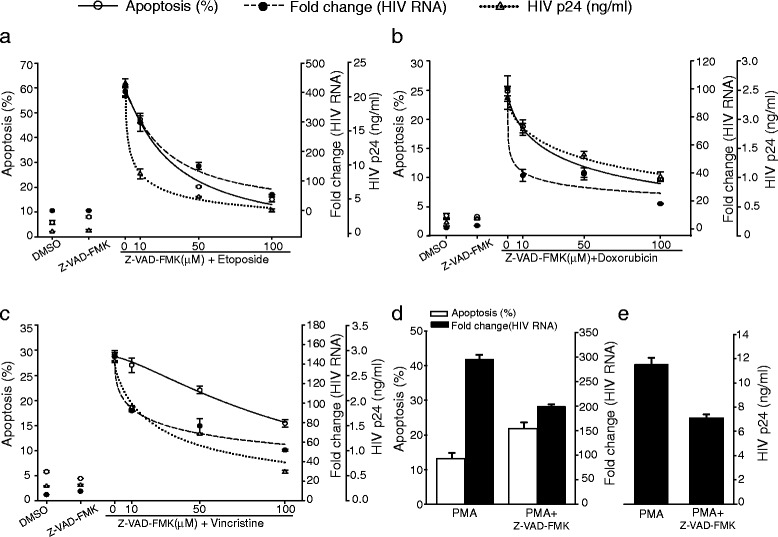
Apoptosis-triggered HIV-1 activation is caspase dependent. U1 cells were pre-treated with increasing concentrations (0-100 μM) of the pan-caspase inhibitor, Z-VAD-FMK for 2 h. U1 cells were treated with pro-apoptotic agents; (**a**) etoposide, (**b**) doxorubicin and (**c**) vincristine to induce apoptosis for 36 h. Cells were stained with Annexin V and 7-AAD and assayed for apoptosis (solid line) using flow cytometry. HIV-1 activation was assessed by quantification of unspliced viral RNA using qRT-PCR and represented as fold change compared cells treated with DMSO-containing medium without cytotoxic agents (dashed line). Culture supernatants were assayed for virus released by determining amount HIV p24-antigen (ng/ml) (dotted line) p24 ELISA. Treatment with all cytotoxic agents led to apoptosis-mediated activation of HIV-1 in U1 cells, which was blocked in a dose-dependent manner by the caspase inhibitor, Z-VAD-FMK. (**d** and **e**) Cells were treated with PMA as a positive control for HIV-1 activation through the conventional pathway. The data represented are the mean ± standard deviation (n = 3)

Caspases play an essential role in mediating the apoptotic cascade. To determine whether caspase activity was necessary for HIV-1 activation by cytotoxic agents and apoptosis, we performed experiments with the general pan-caspase inhibitor, Z-VAD-FMK. We pre-treated U1 cells with increasing concentrations of Z-VAD-FMK (0–100 μM) for 2 h and induced apoptosis with etoposide (Fig. 9a), doxorubicin (Fig. 9b) and vincristine (Fig. 9c). We evaluated apoptosis by Annexin-V staining and measured viral activation by assaying for HIV-1 RNA and p24 production. We observed that pre-treatment with the general caspase inhibitor significantly reduced drug-induced apoptosis and subsequent HIV-1 replication in a dose-dependent manner when compared to untreated cells, suggesting a relationship between apoptosis and HIV-1 activation (Fig. 9 a–c).

**Fig. 10:**
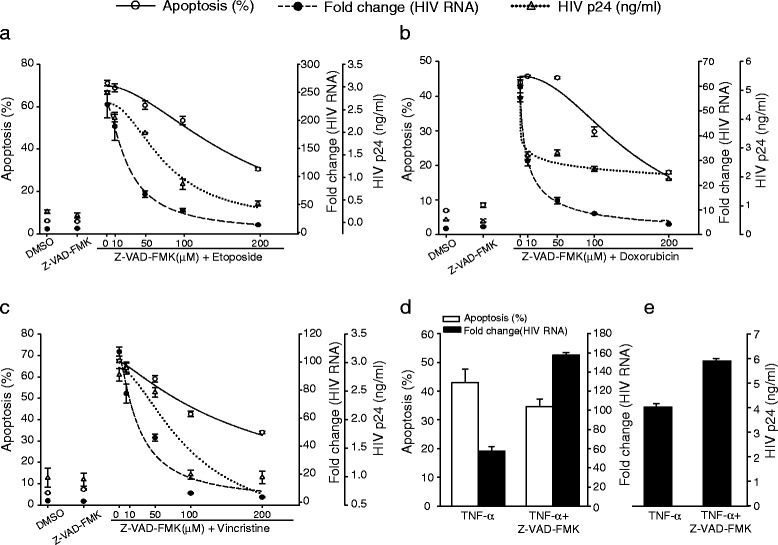
Inhibition of caspase activity and apoptosis-triggered HIV-1 activation in ACH-2 cells. ACH-2 cells were pre-treated with increasing concentration (0–200 μM) of pan-caspase inhibitor, ZVAD-FMK for 2 h prior induction of apoptosis by (**a**) etoposide, (**b**) doxorubicin and (**c**) vincristine. Cells were harvested 36 h post treatment and stained with Annexin V and 7-AAD to assay for apoptosis (solid line) by flow cytometry. HIV-1 unspliced viral RNA was quantified using qRT-PCR and HIV-1 activation was represented as fold change compared to control treatment (dashed line). ACH-2 cells were treated with TNF-α to induce viral replication through the conventional pathway as positive control. Apoptosis-mediated release of HIV-1 virions in culture supernatants was determined by ELISA for HIV-1 p24 antigen (dotted line). (**d** and **e**) The levels of apoptosis (white bars), HIV-1 activation (black bars) and (**e**) virus production followed by TNF-α treatment was determined as in panel A–C. Cells were also pretreated with Z-VAD-FMK (100 μM) for 2 h, followed by TNF-α treatment to determine whether caspase inhibition reduces HIV-1 activation triggered by conventional pathway. Results are pooled from three independent experiments ± SD

We also evaluated the association between apoptosis and activation of HIV-1 replication in lymphocytic cells. We pre-treated ACH-2 cells with Z-VAD-FMK (0–200 μM) and induced apoptosis as described above (Fig. 10). The data obtained in lymphocytic cells, ACH-2, confirmed the results we obtained using U1 cells. Pre-treatment of the cells with Z-VAD-FMK prior to activation by PMA or TNF-α failed to completely block HIV-1 activation, showing that canonical NF-κB-mediated HIV-1 activation is neither apoptosis- nor caspase activation-dependent. In contrast, Z-VAD-FMK treatment prior to activation of ACH-2 cells by TNF-α modestly increased production of HIV-1 RNA and protein, presumably because the Z-VAD-FMK provided some protection against apoptosis occurring in the cells that accompanied conventional HIV-1 replication, enabling production of additional HIV-1 RNA and protein (Fig. 10 d–e) [65, 66]. Overall, the data suggests that apoptosis-mediated activation of HIV-1 replication by cytotoxic drugs requires caspase activity.

### Caspase dependence of apoptosis-triggered HIV-1 activation

**Fig. 11:**
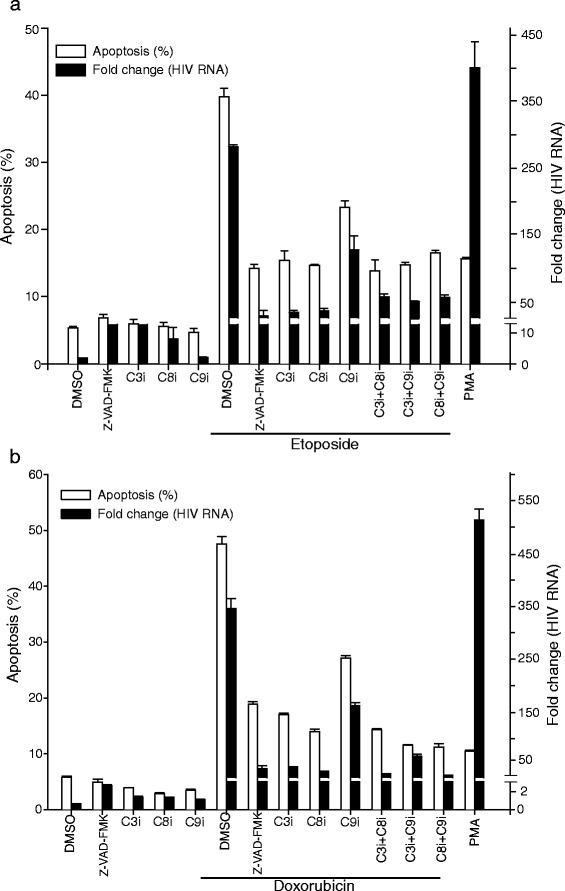
Effects of selective caspase-3 and caspase-8 inhibition on apoptosis induced by cytotoxic agents and associated HIV-1 replication. U1 cells were pre-incubated with in medium with added DMSO alone or with 100 μM of caspase-3 inhibitor (C3i, ZDEVD-FMK), caspase-8 inhibitor (C8i, ZIETD-FMK) and caspase-9 inhibitor (C9i, ZLEHD-FMK) alone or in combination for 2 h. After incubation with the caspase inhibitors, the U1 cells were treated with (**a**) etoposide or (**b**) doxorubicin to induce apoptosis. Apoptosis was assessed after 36 h by Annexin-V-FITC staining and flow cytometry. Total RNA was extracted from each sample and HIV-1 unspliced RNA was quantitated by real-time PCR TaqMan assay (Methods and Materials). White bar represents percent apoptosis and black bar represents fold increase in HIV-1 unspliced RNA compared to DMSO treated cells. Mean values and SD of three independent experiments are shown

The experiments with Z-VAD-FMK (Figs. 9 and 10) showed that caspase activity was necessary for HIV-1 activation following treatment of latently infected cells by cytotoxic agents. We then conducted additional experiments using specific caspase inhibitors to further confirm that caspases were necessary for HIV-1 activation associated with apoptosis and to identify requirements for caspase-associated HIV-1 activation. We treated U1 cells with specific caspase inhibitors alone or in combinations (see Fig. 11 legends) before treating the cells with etoposide (Fig. 11a) and doxorubicin (Fig. 11b). We found that the inhibitors of caspase-3 (Z-DEVD-FMK) and caspase-8 (Z-IETD-FMK) reduced apoptosis and HIV-1 replication following treatment with etoposide and doxorubicin. We found that the caspase-9 inhibitor (Z-LEHD-FMK) also produced modest inhibition of apoptosis and subsequent HIV-1 replication, but did not inhibit activation as effectively as we observed with the caspase-3 and -8 inhibitors. When we treated cells with combinations of the different caspase inhibitors prior to treating the cells with the cytotoxic agents, we observed that the inhibition of either caspase-3 or -8 reduced apoptosis and HIV-1 activation, and that the effect was greater than that observed with the caspase-9 inhibitor (Fig. 11). The data suggest that caspase-3 and -8 are necessary for the activation of latent HIV-1 in association with apoptosis.

### Caspase-3 and -8 activities are sufficient for HIV-1 activation

**Fig. 12:**
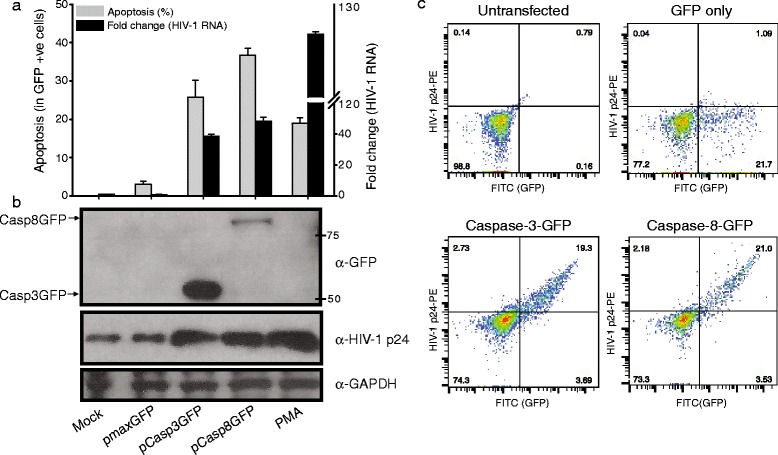
Overexpression of caspase-3 and caspase-8, induction of apoptosis in HIV-1 latently infected cells and activation of HIV-1 replication. (**a**) U1 cells were transfected with pmaxGFP or pCasp3GFP or pCasp8GFP construct were subjected to Annexin V staining followed by flow cytometry, gating on GFP-positive cells, at 36 h post transfection (grey bars). HIV-1 activation was assessed by quantification of unspliced viral RNA using qRT-PCR and represented as fold change compared to DMSO treated cells (black bars). The data are shown as mean ± standard deviation (n = 3). (**b**) Whole cell lysates obtained from transfected cells were subjected to immunoblotting using anti-GFP, anti-HIV p24 and anti-GAPDH antibodies. Anti-GFP used in this experiment is raised against GFP from *Aequorea victoria* while GFP expressed from pmaxGFP® (Lonza) is from *Pontellina plumata*, therefore antibody could not recognize GFP in pmaxGFP® transfected cell lysate. (**c**) Transfected U1 cells were fixed and permeabilized using BDcCyto Fix/Perm solution and stained with HIV-1 p24 antibody (PE conjugated), samples were analyzed using flow cytometry. Upper panel represents untransfected and GFP alone transfected cells while lower panel shows data of caspase-GFP transfected cells

Given that caspase-3 and -8 inhibition are associated with decreases in HIV-1 activation following treatment with cytotoxic drugs, suggesting that these caspases are necessary for the HIV-1 activation response, we wanted to establish whether these activated caspases would be sufficient to initiate HIV-1 replication. We transfected U1 cells with plasmids that express functional activated caspase-3 (pcasp3-Wt-GFP) [67] and caspase-8 (pEGFP-N1-Caspase-8) [68] as GFP fusion proteins, along with a plasmid that expresses only GFP unfused to any other protein, pmaxGFP (Lonza), as negative control. We used treatment with PMA as a positive activation control. After transfection, we examined the cells for Annexin-V staining and GFP expression by flow cytometry. We found that apoptosis, as judged by Annexin-V positivity, accompanied GFP expression in the cells transfected with the caspase-GFP fusion plasmids, the HIV-1 RNA produced by these cells, and HIV-1 p24 released by the cells (Fig. 12). These data indicate that activated caspase-3 or caspase-8 is sufficient to activate HIV-1 from latency.

Since the apoptotic agents or active caspases might have activated HIV-1 expression through a process indirectly related to apoptosis that is *via* some bystander effect, an incidental triggering of viral replication in cells exposed to signaling factors released by cells undergoing apoptosis, we tested whether HIV-1 activation is apoptosis-specific phenomenon. We transfected U1 cells with plasmids expressing caspase-GFP fusion proteins and used flow cytometry to determine whether expression of the HIV-1 p24 protein was produced in the GFP-expressing cells or in all the cells generally. We found that almost all (~90 %) of the cells expressing either caspase-3 or caspase-8 GFP fusion proteins also expressed HIV-1 p24 protein (Fig. 12c, lower panel), compared to control cells that had not been transfected with any plasmid and cells transfected with the control plasmid that expressed only GFP that was not fused to an activated caspase protein. Taken together, these data suggest that activation is directly related to caspase expression and apoptosis and is not a bystander phenomenon.

## Discussion

Many viruses, including HIV-1, have evolved functions that inhibit host cell apoptosis during viral replication (reviewed in [69, 70]). We, and others, recently showed that herpesviruses can apparently sense host cell apoptosis and respond by initiating an alternative, rapid or disordered program of viral replication that does not use the same regulatory proteins used by the conventional replication pathway, and produces virions of decreased infectivity [52–55]. Our current study suggests that HIV-1 can also sense host cell apoptosis and initiate replication in response. The ability to sense when the host cell is about to undergo apoptosis and respond by initiating viral replication would seem to offer viruses capable of long term latency a substantial evolutionary advantage: otherwise any viruses living latently in host cells that underwent apoptosis would not reproduce. The existence of conceptually similar abilities to sense and respond to host cell apoptosis by two very different viruses capable of long term latency may suggest that any virus capable of long term latency would have likely evolved some kind of analogous alternative apoptosis-triggered replication pathway.

How activated caspases trigger replication of latent virus – HIV-1 or herpesviruses – is not completely understood, but one plausible hypothesis would be that caspase-mediated cleavage of a viral or host factor converts an inert protein into a potent transactivator. For herpesviruses, there is evidence that some viral proteins are the targets of caspase activity. For example ICP-22, a protein of HSV-1 involved in maintaining latency has been shown to be cleaved by capase-3 [71]. While the sensing mechanisms for both herpesviruses and HIV-1 involve activated caspases, the downstream details of the mechanisms are likely to be very different given the great differences in the viruses, with host cell proteins much more likely to be involved in the HIV-1 sensing mechanism due to its much smaller genome size and complement of encoded proteins.

There has been ongoing interest in the relationships between apoptosis and HIV-1 replication and pathogenesis beyond the important studies that describe how HIV-1 inhibits host cell apoptosis during viral replication. Treatment of latently infected host cells with Z-VAD-FMK enhances production of HIV-1 replication when viral replication in those cells is initiated by treatment with TNF-α, but no direct effect on the virus was observed [65]; the increase in HIV-1 production likely resulted from Z-VAD-FMK inhibiting host cell apoptosis during the process of viral replication, protecting that process, like the helpful effects of HIV-1’s own anti-apoptotic activities. We also observed that Z-VAD-FMK could enhance TNF-α’s capability to induce latent HIV-1 compared to TNF-α alone Fig. 10 (d and e). Studies of the relationship between HIV-1 latency and host cell apoptosis also have interesting clinical implications. Some investigators have proposed research aimed at devising ways to specifically induce apoptosis in cells latently infected with HIV-1 as a way of attacking and depleting the reservoir of cells latently infected with HIV-1, as a way of effecting a cure for HIV infection (reviewed in [10, 72]). If such strategies are used, given our findings, it may be helpful to consider that treating patients with agents to induce apoptosis in an effort to deplete the latent reservoir may lead to the activation of HIV-1 replication and the production of large amounts of virus.

The observations that HIV-1 can sense host cell apoptosis, notably when triggered by cytotoxic drugs used as cancer chemotherapy and bone marrow transplant conditioning agents, suggest some additional clinical implications of our findings. The most dramatic reductions in the HIV-1 latent reservoir have been observed in patients treated with highly cytotoxic chemotherapy and bone marrow conditioning regimens. The reservoir reduction in these patients has conventionally been attributed to the destruction of the cells that constitute the long lived reservoir of latently infected cells, perhaps with some additional contribution of a graft *vs.* infected cell immune response in the bone marrow transplant patients. Our data suggests that activation of latent virus by host cell apoptotic signals might also contribute to reservoir reduction, particularly when the infection of new cells is blocked.

A detailed understanding of the pathways that mediate HIV-1 apoptosis sensing and replication might lead to the identification of new, targetable elements of those pathways. Given the potent activation that we observed in the latently infected cells, particularly those arrested in G_1_, and the substantial reductions in the HIV reservoirs observed in patients treated with cytotoxic drugs, targeting these points may be particularly effective at activating HIV, even in cells in which HIV previously available activating agents were not effective. While trying to attack and deplete the reservoir of latently infected cells with cytotoxic agents is clearly impractical, studies of the pathways that mediate apoptosis-initiated HIV replication might lead to the identification of new activating agents, perhaps including agents that do not trigger apoptosis.

## Methods

### Cell lines

HIV-1 latently infected model cell lines U1 [73], derived from the U937 promonocytic cell line and ACH-2 [74] derived from the A3.01 T lymphocytoid cell line were obtained from NIH AIDS Research and Reference Reagents Program. The cells were maintained in RPMI 1640 (Invitrogen) medium supplemented by 10 % fetal calf bovine serum (FBS, Hyclone), 1 % L-glutamine (Sigma Aldrich), penicillin (100 IU/ml) and streptomycin (100 μg/ml) (Sigma Aldrich).

### Induction and determination of apoptosis

Cytotoxic drugs etoposide (VP-16), fludarabine phosphate, doxorubicin and vincristine (all from Sigma-Aldrich), dissolved in dimethyl sulfoxide (DMSO, Sigma-Aldrich) were used to treat cells. To expose the cells to the cytotoxic drugs, the cells were collected, washed with serum-free medium, resuspended in complete medium with final cell concentration of 0.25 × 10^6^ cells per ml. Cells were exposed to the drugs at final concentrations as mentioned in figure legends. We used phorbol myristyl acetate (PMA) (25 ng/ml; Sigma Aldrich) and tumor necrosis factor-α (TNF-α) (20 ng/ml; Invitrogen) as positive control HIV-1 inducing agents. 36 h post treatment, cultures were divided in two parts, one for RNA extraction and p24 assay in the supernatant and another for Annexin V staining to measure apoptosis.

Cells were collected in 1.5 ml microcentrifuge tubes by centrifugation at 1200 rpm for 5 min. To assay for apoptosis, the cells were washed with cold 1X phosphate buffered saline (PBS) and resuspended in 100 μl of 1X binding buffer (BD Biosciences). We added 5 μl of Annexin V-FITC (BD Biosciences) to the samples and incubated the cells for 20 min in the dark at room temperature before mixing with 400 μl of 1X PBS supplemented with 7-AAD (7-Aminoactinomycin D) (10 μg/ml). The cells were analyzed using a FACSCanto-II flow cytometer (BD Biosciences). Untreated cells were used to establish forward- and side- scatter gates. Data was analyzed with FACSDiva software (BD Biosciences).

### Apoptosis inhibition

The general caspase inhibitor, Z-VAD-FMK (Enzo Life Sciences) and specific caspase inhibitors, Z-DEVD-FMK (caspase-3 inhibitor), Z-IETD-FMK (caspase-8 inhibitor) and Z-LEHD-FMK (caspase-9 inhibitor) (all from BD Bioscience) were reconstituted in DMSO (Sigma Aldrich) to a stock concentration of 10 mM. Cells were collected, washed with PBS, resuspended in culture medium at final concentration of 0.25 × 10^6^ cells per ml. For apoptosis inhibition, cells were pre-treated with specific inhibitors of caspases (0-200 μM) for 2 h before inducing apoptosis by cytotoxic drugs. PMA (25 ng/ml) or TNF-α (20 ng/ml) were used as positive control HIV-1 activators. The treated cells were incubated for 36 h in 5 % CO_2_ and 37 °C in humidified atmosphere. After 36 h cultures were divided and collected in two parts, one for RNA extraction and another for Annexin-V staining.

### Assays for HIV-1 RNA

RNA was extracted from the cell pellet using the RNeasy Mini Kit (Qiagen) according to the manufacturer’s protocol. RNA was quantified using a NanoDrop HD-1000 Spectrophotometer (Thermo). Only samples with 260/280 ≥ 2 absorbance ratios were used. We used 500 ng of total RNA per sample for reverse transcription reactions using iScript^TM^ Reverse Transcription Supermix for qRT-PCR (Bio-Rad) according to manufacturer’s protocol. Briefly, the RNA was mixed with 4 μl of 5 X iScript RT Supermix and total reaction volume was made-up to 20 μl by adding RNase-free water (Qiagen). For negative control or no reverse transcriptase control (NRTC) we used reaction mixture made by mixing RNA, water and 4 μl of 5 X iScript no-RT supermix. The reactions were incubated for 5 min at 25 °C for primer annealing, 60 min at 42 °C for reverse transcription and then at 85 °C for 5 min for enzyme inactivation. The cDNA reactions were diluted 10 fold and 2 μl of diluted cDNA was used in real time PCR reactions. For the qPCR reactions we used TaqMan master mix system (Applied Biosystems) and TaqMan probes specific for HIV-1 late RNA (unspliced RNA) (IDT) and human GAPDH (Applied Biosystems). The sequences of primer sets used to amplify HIV-1 unspliced RNA were 5′-ATAATCCACCTATCCCAGTAGGA GAAAT-3′ (SK38) 5′-TTTGGTCCTGTGCTTATGTCCAGAATGC (SK39) [75]. A FAM-TAMRA-labeled probe 5′-ATCCTGGGATTCAATAAAATAGTAGAGATGTATAGCCCTAC-3′ was used for quantitation of late viral RNA species [75]. The thermal cycling conditions were 50 °C for 2 min and an initial denaturation at 95 °C for 15 s followed by 40 cycles at 95 °C for 15 s and 60 °C for 60 s using the Applied Biosystems 7500 Fast Real Time PCR detection system. All reactions were performed in 20 μl final volume, with human GAPDH was used as endogenous control and NRTC as negative control. The amount of PCR product was determined by the comparative 2^-ΔΔCt^ method [76], with each sample normalized to human GAPDH and expressed as a fold-increase versus untreated controls.

### HIV-1 p24 protein detection by flow cytometry

To measure HIV-1 p24 protein expression, cells were fixed and permeabilized with BD Cyto Fix/Perm kit (BD Biosciences), washed with PBS containing 1 % FCS, and stained with anti-HIV-1 p24 phycoerythrin mAb KC57 (Beckman Coulter) at 1:500 dilution. Isotype-matched mAbs were used as negative controls. Samples were analysed with (BD Biosciences) and FloJo software.

### Induction of G_1_-phase arrest and cell cycle analysis by flow cytometry

Simvastatin, a potent G_1_-phase blocker was purchased from Sigma and dissolved in DMSO. Cells in a density of 0.25 × 10^6^ /ml were serum starved for 12 h and then treated with Simvastatin (5 μM) for another 18 h. Cells were collected and fixed by resuspending them in 0.5 ml of 100 % ethanol (ice-chilled) for 30 min on ice and then centrifuged at 1500 rpm for 10 min and washed in ice-cold PBS + 1 % serum. The cell pellets were resuspended in 0.5 ml PBS + 1 % serum containing 50 μg/ml propidium iodide (BD Biosciences) and 100 μg/ml RNase (Invitrogen), incubated at 37 °C for 30 min, and then analyzed using a FACSCalibur flow cytometer (Becton Dickinson)

### Determination of HIV-1 virion infectivity

The 1G5 cell line, a Jurkat derivative containing a stably integrated HIV-LTR-luciferase construct [62], was obtained from NIH AIDS Research and Reference Reagents Program and maintained in 10 % FBS (Hyclone), 1 % L-glutamine (Sigma Aldrich), penicillin (100 IU/ml) and streptomycin (100 μg/ml) (Sigma Aldrich). For the infectivity assay, cells were seeded at 0.2 × 10^6^ cells per well in 0.5 ml of RPMI 1640 supplemented with 10 % fetal bovine serum and Polybrene (4 μg/ml, Sigma). Cells were left untreated or treated with 3′-Azido-3′-deoxythymidine, 5 μM (AZT, Sigma Aldrich) for 2 h prior to infection with HIV-1 virions (200 ng) normalized according to p24 amounts present in cell-free culture supernatants of drug-treated ACH-2 cells. 1G5 cells were incubated with virions in 0.5 ml of complete medium supplemented for 4 h with continuous rocking at 37 °C in the presence of Polybrene (4 μg/ml) (Sigma Aldrich) and AZT (5 μM). The cells were washed 2 times with serum-free media to remove unbound HIV-1 and re-suspended in 2 ml fresh complete media containing AZT (5 μM) and incubated for an additional 44 h at 37 °C in humidified CO_2_-incubator. Post 44 h incubation, cells were harvested and lysed using 200 μl of 1X cell culture lysis reagent (CCLR, Promega). Luciferase assays were performed on clarified lysate using BrightGlo Luciferase Assay System (Promega) according to manufacturer’s instructions.

### Treatment of cells supporting active viral replication with cytotoxic agents

Jurkat T-cells were obtained from NIH AIDS Research and Reference Reagents Program and maintained in 10 % FBS (Hyclone), 1 % L-glutamine (Sigma Aldrich), penicillin (100 IU/ml) and streptomycin (100 μg/ml) (Sigma Aldrich). For infection, cells were washed, seeded at 0.25 × 10^6^ cells per well in 0.5 ml of RPMI 1640 supplemented with 10 % fetal bovine serum and Polybrene (4 μg/ml, Sigma) and infected with HIV-1 virions present in cell-free culture supernatants of TNF-α treated ACH-2 cells. Four hours post infection cells were washed 2 times with RPMI to remove the unbound virus and resuspended in 2 ml of fresh complete medium. Apoptosis was induced by treating the infected cells with etoposide (0.5 μM), doxorubicin (0.5 μM), vincristine (1 nM) and fludarabine phosphate (1 μM). Induction of apoptosis in these cells was assessed by flow cytometry, and viral gene expression was determined by qRT-PCR, as described above.

### Transfection of cells with plasmids expressing caspase-3—GFP and caspase-8-GFP fusion proteins

U1 cells were transfected with plasmids, pcasp3-Wt-GFP (simplified to pCasp3GFP in the figures and text) a generous gift from Shinji Kamada, Biosignal Research Center, Kobe University [67] and pEGFP-N1-caspase 8 (simplified to pCasp8GFP in the figures and text) a kind gift from Eyal Gottlieb, The Beatson Institute for Cancer Research, Glasgow, UK [68]. pmaxGFP® (Lonza) was used for GFP-only control and pUC19 was used as a negative control (Mock). Cells were seeded in 12-well plates, and 2 h prior to transfection, the medium was replaced by RPMI 1640 medium without FBS and antibiotics. Lipofectamine 3000 (Life Technologies) was mixed with 100 μl Opti-MEM I medium (Life Technologies) at a 1:50 dilution and incubated for 10 min at RT. This mixture was then complexed with 1 μg of plasmids and 2 μl of reagent P diluted in Opti-MEM and incubated at RT for 25 min. The complex was added to the cells, and the plates were gently rocked at 37 °C for 5 h. After 5 h, medium was replaced with RPMI with 10 % FBS and cells were incubated for 36 h. Cells were also treated with PMA (25 ng/ml) as a positive control for HIV-1 activation through the conventional pathway and etoposide (10 μM) as a positive control for apoptosis induced by a cytotoxic agent. After incubation, cells were harvested and one aliquot was evaluated for Annexin-V-APC staining, another aliquot was used for RNA isolation, as described above, and a third aliquot was used for protein isolation. For the protein isolation, the cell pellet was lysed in TN-lysis buffer (20 mM Tris-Cl, 150 mM NaCl, 1 mM EDTA, 0.5 mM PMSF, 0.5 % NP-40 and 1X protease inhibitor) by incubating for 45 min on ice with intermittent shaking. The protein present in the clarified lysate was estimated using Bradford reagent (BioRad) according to manufacturer’s instruction and 40 μg of protein in the lysate was used for immuno-blotting. Monoclonal antibodies against GFP (Cell Signaling) and GAPDH (HRP-labeled) (Abcam) were used at 1:2000 dilution. HIV-1 p24 was detected by using 1:100 diluted supernatant fluid from the anti-HIV p24 hybridoma 183-H12-5C [77], obtained from the NIH AIDS Research and Reference Reagent Program. Peroxidase-conjugated anti-mouse antibody (Santa Cruz) was used as secondary antibody to detect GFP and HIV-1 p24 proteins by chemiluminiscent immunoblotting detection reagent (Amersham Biosciences).

### HIV-1 p24 Capture ELISA

HIV-1 p24 antigen was quantified in drug-treated cell culture supernatant by performing a p24 antigen enzyme linked immunosorbent assay (ELISA) using commercially available ELISA kit (p24 HIV antigen ELISA kit, Perkin Elmer) according to manufacturer’s protocol.

### Statistics

Values represent the mean ± SD of at least three independent experiments. Correlation between apoptosis and HIV-1 activation was calculated by performing the Spearman rank correlation test using SigmaPlot 11.0. Data curve fitting (Gompertz) and non-linear regression statistical analyses were accomplished using SigmaPlot 11.0 software.
